# Biodegradable Micelles for NIR/GSH-Triggered Chemophototherapy of Cancer

**DOI:** 10.3390/nano9010091

**Published:** 2019-01-11

**Authors:** Chuan Zhang, Yuzhuo Wang, Yue Zhao, Hou Liu, Yueqi Zhao, Xiangwei Li, Quan Lin

**Affiliations:** 1 State Key Laboratory of Supramolecular Structure and Materials, College of Chemistry, Jilin University, Changchun 130012, China; 15943022539@163.com (C.Z.); 18204319457@163.com (Y.Z.); liuhou17@mails.jlu.edu.cn (H.L.); 18844191429@163.com (Y.Z.); 2 Department of Orthodontics, School of Stomatology, Jilin University, 1500 Qinghua Road, Changchun 130021, China; 13104467667@163.com; 3 School of Stomatology, Jilin University, Changchun 130041, China

**Keywords:** photothermal therapy, photodynamic therapy, biodegradable, stimuli-responsive drug delivery systems, cypate

## Abstract

The chemotherapy of stimuli-responsive drug delivery systems (SDDSs) is a promising method to enhance cancer treatment effects. However, the low efficiency of chemotherapy drugs and poor degradation partly limit the application of SDDSs. Herein, we report doxorubicin (DOX)-loading mixed micelles for biotin-targeting drug delivery and enhanced photothermal/photodynamic therapy (PTT/PDT). Glutathione (GSH)-responsive mixed micelles were prepared by a dialysis method, proportionally mixing polycaprolactone-disulfide bond-biodegradable photoluminescent polymer (PCL-SS-BPLP) and biotin-polyethylene glycol-cypate (biotin-PEG-cypate). Chemically linking cypate into the mixed micelles greatly improved cypate solubility and PTT/PDT effect. The micelles also exhibited good monodispersity and stability in cell medium (~119.7 nm), low critical micelles concentration, good biodegradation, and photodecomposition. The high concentration of GSH in cancer cells and near-infrared light (NIR)-mediated cypate decomposition were able to achieve DOX centralized release. Meanwhile, the DOX-based chemotherapy combined with cypate-based NIR-triggered hyperthermia and reactive oxygen species could synergistically induce HepG2 cell death and apoptosis. The in vivo experiments confirmed that the micelles generated hyperthermia and achieved a desirable therapeutic effect. Therefore, the designed biodegradable micelles are promising safe nanovehicles for antitumor drug delivery and chemo/PTT/PDT combination therapy.

## 1. Introduction

The emergence of photothermal therapy (PTT) and photodynamic therapy (PDT) has gotten a satisfactory therapeutic effects, and so researchers have attempted to combine PTT and PDT to change therapeutic strategies and improve treatment effects in cancer therapy [1,2,3,4]. Generally, PTT involves energy transformation from near-infrared light (NIR) photons into hyperthermia, which is more poisonous to cancer cells than normal cells [5]. PDT therapy is based on the generation of reactive oxygen species to kill cancer cells when photosensitizers are exposed to various wavelengths of light in the presence of dissolved oxygen [6]. These synergic therapy methods have achieved efficient antitumor activity through the phototoxicity of PTT or PDT. In addition, chemotherapy based on stimuli-responsive drug delivery systems (SDDSs) have been implemented in extensive research and have been combined with phototherapy [7,8,9,10,11]. Various sensitive functional groups (e.g., disulfide, hydrazide, and imidazole) have been widely introduced into SDDSs to achieve controllable drug release and cancer therapy [12,13,14,15,16]. Among them, the reductive glutathione (GSH), with a discrepant concentration gradient between the extracellular fluid (2–10 μM) and the tumor intracellular fluid (2–10 mM), can allow SDDSs to quickly release their payload through the cleavage of disulfide bonds to achieve chemotherapy [17,18,19]. Although SDDSs-based chemotherapy has been widely investigated in cancer treatment, the low efficiency of chemotherapy drugs and poor degradation partly limit the application of SDDSs, greatly reducing therapy efficiency and leaving behind potential toxicity of nondegradable materials [20,21,22]. Therefore, improving treatment efficiency through PTT/PDT and carrying out degradable materials may be a prospective choice for cancer treatment.

So far, PTT and PDT therapy have been performed with several organic NIR dyes and various inorganic materials, including indocyanine green (ICG), upconversion nanoparticles (UCNPs), or gold nanorods, acquiring many achievements [23,24,25]. Cypate, a derivative of ICG, possesses similar properties (i.e., the generation of reactive oxygen species, biocompatibility, and a high extinction coefficient) to ICG [26]. Furthermore, cypate decomposes into smaller fragments upon NIR irradiation, and the hydrophobicity transforms from strong to weak [27]. Two reactive carboxylic acid groups from cypate also ensure that the cyanine dye can be introduced to SDDSs. Based on these properties of cypate, introducing cypate to micelles as their hydrophobic core and enhancing payload release upon NIR light irradiation is available. Many previous studies have focused on the use of cypate for single phototherapy (i.e., PDT or PTT) or optical imaging by physical assembly or chemical conjunction of the dye into various types of nanovehicles such as silica nanochannels, micelles, and UCNPs, while the therapy effect and nondegradable materials still hindered SDDSs application [28,29,30]. Therefore, the strategy of combining cypate and biodegradable polymers displays unsurpassed superiority for chemophototherapy.

**Scheme 1:**
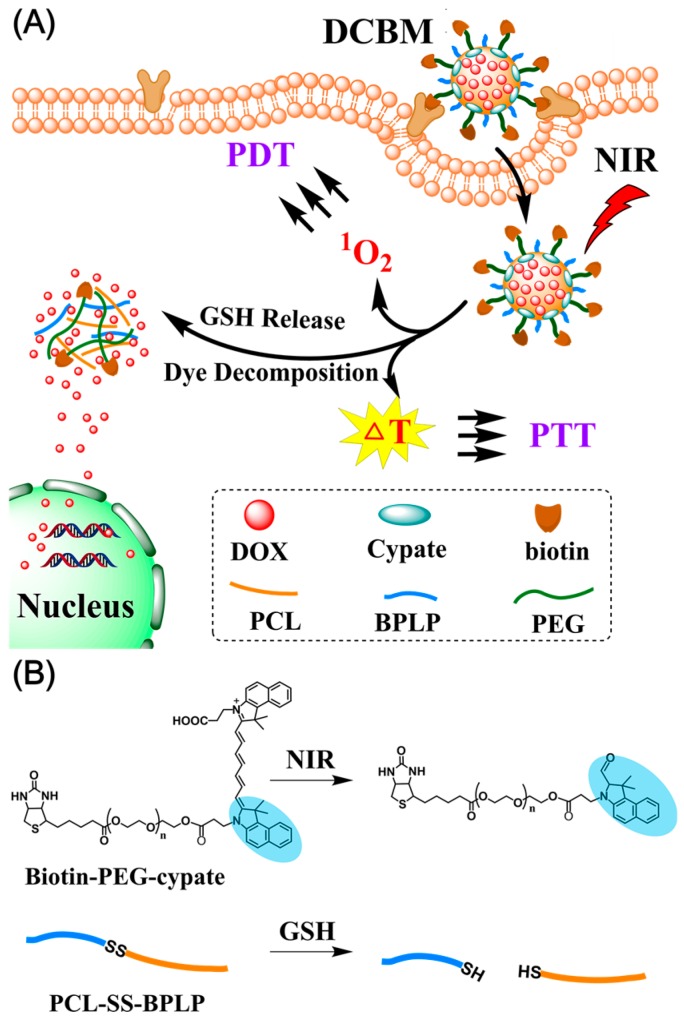
(**A**) Glutathione (GSH)/light-induced release of an anticancer drug (doxorubicin (DOX)) from degradable polymer micelles (doxorubicin/cypate-loading biodegradable micelles (DCBMs)) and photothermal/photodynamic therapy (PTT/PDT therapy); (**B**) decomposition procedure of biotin-polyethylene glycol-cypate (biotin-PEG-cypate) under near-infrared light (NIR) irradiation and degradation of polycaprolactone-disulfide bond-biodegradable photoluminescent polymer (PCL-SS-BPLP) in the GSH environment.

Herein, we developed doxorubicin/cypate-loading biodegradable micelles (DCBMs) for cancer-targeted delivery and synergistic chemophototherapy, chemically linking the hydrophobic cypate into micelles to improve its solubility and using biodegradable photoluminescent polymers (BPLPs) as the skeleton. DCBMs exhibited an excellent biodegradation profile, photodegradability, and superior photocytotoxicity. Previous work confirmed the degradation products of BPLP were less toxic [31]. As illustrated in Scheme 1, the designed micelles could accumulate in tumor tissue by biotin-avidin-mediated cancer cell membranes targeting and further uptake through endocytosis [32,33]. Under the exposure of NIR, DCBM could rapidly generate reactive oxygen species (ROS) and hyperthermia in the cytoplasm to induce cell death and apoptosis. Meanwhile, the high concentration of glutathione in the cancer cell and NIR-mediated cypate decomposition also accelerated doxorubicin (DOX) release in the tumor area, resulting from a break in the amphipathic balance in DCBMs. Thus, hyperthermia, ROS, and accumulated DOX in cytoplasm trinitarianly produced satisfactory capability of cell killing. The designed biodegradable DCBM is a promising safe nanovehicle for antitumor drug delivery and synergistic chemo/PTT/PDT combination therapy.

## 2. Materials and Methods

### 2.1. Materials

Cysteine, citric acid, PEG200, ε-caprolactone, pyrene, 1, 3-disphenylisobenzofuran (DPBF), 1-(3-dimethylaminopropyl)-3-ethylcarbodiimide hydrochloride (EDC), N-hydroxysuccinimide (NHS) and benzylalcohol were all obtained from Aladdin (Shanghai, China). Enzyme 435, 4-nitrophenyl chloroformate (NPC), cystamine dihydrochloride, and glutathione were provided by J&K Scientific LTD (Beijing, China). Doxorubicin hydrochloride (DOX·HCl) was supplied by Dalian Meilun biotechnology Co., LTD (Dalian, China). Biotin-PEG2000-NH_2_ (Mw = 2000 Da) was purchased from Shanghai Ponsure Biotechnology Co. Ltd (Shanghai, China). 2′, 7′-dichlorodihydrofluorescein diacetate (DCFH-DA), Annexin V-FITC, propidium iodide (PI), and Hochest 33342 were purchased from Beyotime biotechnology Co., LTD (Shanghai, China). Cypate was synthesized referring to previous work. All other chemicals were obtained from Beijing Chemical Works (Beijing, China) and were used without further purification.

### 2.2. Preparation of Biotin-Polyethylene Glycol-Cypate (Biotin-PEG-Cypate)

**Scheme 2:**
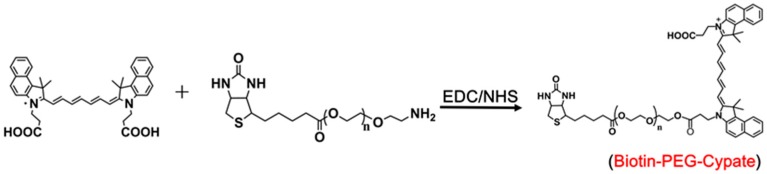
Synthesis route of biotin-PEG-cypate.

Biotin-PEGylated-cypate was synthesized by chemically linking cypate with biotin-PEG2000-NH_2_ via EDC/NHS reaction (Scheme 2). In brief, EDC (100 μmol) and NHS (100 μmol) were added to cypate (75 μmol) in dichloromethane (DCM, 8 mL). After stirring for 2 h, biotin-PEG2000-NH_2_ (75 μmol) was added into the dichloromethane solution, and the mixture was reacted for 12 h at room temperature in the dark. After evaporating the solvent, the crude product was purified, followed by dispersion with distilled water, followed by filtration and dialysis (the molecular weight cut-off was 1000). ^1^H NMR, ^13^C NMR (300 MHz), elemental analysis, gel permeation chromatography (GPC), and Fourier transform infrared spectroscopy (FTIR) were performed.

### 2.3. Preparation of Cystamine-Terminated Polycaprolactone (PCL-SS-NH_2_)

#### 2.3.1. Synthesis of Monohydroxy-Terminated Polycaprolactone (PCL-OH)

**Scheme 3:**
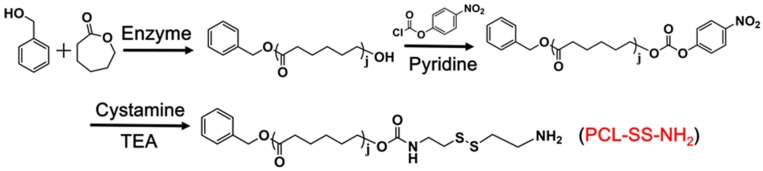
Synthesis route of PCL-SS-NH_2_.

PCLOH was synthesized by the enzymatic ring-opening polymerization of ε-caprolactone (Scheme 3) [34]. In brief, ε-caprolactone (30 mmol) and benzyl alcohol (1 mmol) were added into dry methylbenzene (10 mL), and enzyme 435 (0.144 g) was added. The polymerization reaction was carried out at 70 °C for 12 h. After removing the enzyme 435 and methylbenzene, the product was precipitated three times in cold methanol. The white polymer was dried in a vacuum environment at room temperature for 48 h. The structure of PCL-OH was verified using ^1^H NMR (500 MHz, CDCl_3_) δ 7.36 (t, 5H, C_6_**H**_5_–), 5.13 (s, 2H, C_6_H_5_–C**H**_2_–), 4.08 (t, 2H, –C**H**_2_O–), 2.32 (t, 2H, –C**H**_2_CO–), 1.68 (m, 4H, –C**H**_2_–), 1.40 (m, 2H, –C**H**_2_–).

#### 2.3.2. Synthesis of PCL-SS-NH_2_

Activated PCL polymer was synthesized in two steps (Scheme 3) [35]. First, PCL-OH (0.5 g) and pyridine (0.1 mL) were dissolved in dry DCM (10 mL) under an ice bath condition. Then 3 mL of NPC (0.05 g) solution in DCM was slowly added. The mixture was stirred at room temperature for 3 h. After that, the crude product PCL-NPC was precipitated in cold diethyl ether three times and dried overnight under vacuum. The structure of PCL-NPC was verified using ^1^H NMR (500 MHz, CDCl_3_) δ 8.18–8.38 (4H, C_6_**H**_4_NO_2_), 7.37 (t, 5H, C_6_**H**_5_–), 5.14 (s, 2H, C_6_H_5_–C**H**_2_–), 4.08 (t, t, 2H, –C**H**_2_O–), 2.33 t, 2H, –C**H**_2_CO), 1.67 (m, 4H, –C**H**_2_–), 1.40 (m, 2H, –C**H**_2_–).

Second, cystamine dihydrochloride (1 g) and triethylamine (TEA, 2.4 mL) were dissolved in dry methanol (5 mL). Then 15 mL of PCL-NPC (0.5 g) solution in tetrahydrofuran (THF) was slowly added to obtain a yellow solution. The reaction proceeded for 24 h at room temperature. After removing the solvent and redispersing in distilled water, the filter cake was collected by filtration. The sample was dried under vacuum and precipitated several times until obtaining a white product. The structure of PCL-SS-NH_2_ was verified using ^1^H NMR (500 MHz, CDCl_3_) δ 7.35 (t, 5H, C_6_**H**_5_–), 5.12 (s, 2H, C_6_H_5_–C**H**_2_–), 4.06 (t, 2H, –C**H**_2_O–), 3.41 (2H, –C**H**_2_–), 3.17 (2H, –C**H**_5_–), 2.88 (t, 2H, –C**H**_2_–S–), 2.82 (t, 2H, –C**H**_2_–S–), 2.31 (t, 2H, –C**H**_2_CO), 1.66 (m, 4H, –C**H**_2_–), 1.38 (m, 2H, –C**H**_2_–).

### 2.4. Preparation of Polycaprolactone-Disulfide Bond-Biodegradable Photoluminescent Polymer (PCL-SS-BPLP)

**Scheme 4:**
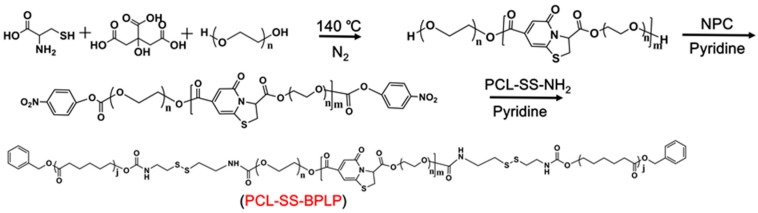
Synthesis route of PCL-SS-BPLP.

PCL-SS-BPLP copolymer was synthesized in two steps (Scheme 4). Step one involved the preparation of a hydroxyl-terminated BPLP prepolymer, as described in other work [31]. PEG200, citric acid, and L-cysteine with a molar ratio of 1.2:1:0.2 were added into a three-necked round bottom flask. Under a nitrogen atmosphere, the mixture was heated to 160 °C until all reactants melted. The temperature of the system was subsequently decreased to 140 °C, and the reaction continued to react for 2.5 h. The obtained BPLP polymer was purified by a dialysis membrane (the molecular weight cut-off was 1000). The structure of BPLP was verified using ^1^H NMR (500 MHz, D_2_O, Figure S1).

In the second step, the BPLP prepolymer (1 g) and pyridine (0.1 mL) were dissolved in 5 mL dry THF. Then a solution of NPC (0.05 g) in 3 mL dry THF was slowly added. The mixture was stirred at room temperature for 12 h. After removing the excessive NPC by dialysis, the sample was slowly added into 10 mL THF solution containing 1 g of PCL-SS-NH_2_. Then, the mixture was stirred at room temperature for another 12 h. Next, the obtained PCL-SS-BPLP polymer was purified by a dialysis membrane (the molecular weight cut-off was 3500) and further filtered to remove the excessive PCL-SS-NH_2_. The structure of the polymers was verified using ^1^H NMR (500 MHz), ^13^C NMR (300 MHz), elemental analysis, gel permeation chromatography (GPC), and Fourier Transform infrared spectroscopy (FTIR).

### 2.5. Preparation and Characterization of Mixed Micelles

All the micelles were prepared using the dialysis method [36]. In brief, DOX·HCl and sodium carbonate were added to dimethyl sulfoxide (DMSO) with a molar ratio of 1:1.05 and stirred for 12 h to remove the hydrochloride. After removing the sodium carbonate, biotin-PEG-cypate and PCL-SS-BPLP with a weight ratio of 1:1 were added to the DMSO solution. The solution was dialyzed against water for 24 h to obtain the DOX/cypate-loading biodegradable micelles (DCBMs). The preparations of cypate-loading biodegradable micelles (CBMs) and DOX-loading biodegradable PCL-SS-BPLP only micelles (DBMs) were similar to the above procedures.

The size distribution and morphology of mixed micelles were determined by dynamic light scattering (DLS) and TEM. The concentration of DOX was measured by a UV-Vis spectrometer at 481 nm. The DOX content of micelles was calculated by the formula: DOX content (% *w*/*w*) = (weight of DOX in micelles)/(weight of DOX in micelles + weight of two polymers) × 100%.

### 2.6. Critical Micelle Concentration (CMC) Measurement

The CMC of mixed micelles was measured using pyrene as a hydrophobic fluorescent probe [37]. The blank micelles solutions in various concentrations were equilibrated with a constant concentration of pyrene (1 × 10^−6^ M) for 24 h in the dark. The CMC was calculated by the variation in the ratio of fluorescent intensity I_373_/I_395_ of pyrene, which was carried out using a fluorescence spectrometer.

### 2.7. In Vitro Degradation of PCL-SS-BPLP

The degradation experiments of PCL-SS-BPLP were done with reference to a previous work with slight changes [38]. In brief, 10 mg of the copolymer was placed in a centrifuge tube containing 2 mL of phosphate-buffered saline (PBS, pH = 7.4). To eliminate photodegradation interference, the copolymer was openly stored at room temperature in a dark environment. Fresh distilled water was added to the tube to ensure a constant volume of 2 mL. The degradation effects were determined by photoluminescence intensity changes.

### 2.8. NIR Irradiation-Triggered Decomposition, Photothermal Effects, and ROS Generation of Mixed Micelles

To measure the photothermal conversion performance of DCBM, 1 mL of DCBM (10 μg/mL of cypate) solution was irradiated for 300 s with a continuous-wave NIR light (808 nm, 1 W/cm^2^). The temperature changes of DCBM during NIR irradiation were recorded using a thermocouple thermometer and infrared and thermal (IR) camera. Light-induced cypate decomposition changes of CBM (10 μg/mL of cypate) with a continuous-wave NIR light (808 nm, 1 W/cm^2^, 600 s) were also recorded using a UV-Vis spectrophotometer.

To measure the photothermal conversion efficiency (η), the free cypate and DCBM dispersion (cypate: 10 μg/mL; solvent: V_water_/V_ethanol_ = 9:1) were irradiated by the NIR light until the system temperature reached a steady state [39]. After the NIR light was turned off, the temperature was cooled to room temperature. The photothermal conversion efficiency was calculated by two equations: (a) η = (*hA* × *ΔT_max_* − *Q_0_*)/(*I* × (1−10*^−A^_λ_*)), (b) *t* = − (*ln(ΔT/ΔT_max_*) × *∑_i_m_i_C_p,i_*)/(*hA*). The *ΔT (ΔT_max_)* is the temperature difference between sample temperature (maximum temperature) and room temperature, *I* is the power of NIR light illuminated on the sample, *m* is the sample mass, *C_p_* is the specific heat of solvent, *hA* is derived from the slope of the cooling period time *t* versus *ln(ΔT/ΔT_max_)* graph, *Q_0_* is the heat induced by the light absorption of the solvent, and *A_λ_* is the absorbance of the sample solution at 808 nm.

For the photodynamic effects measurement, CBM (5 μg/mL of cypate) was evaluated using 1, 3-disphenylisobenzofuran (DPBF) as a singlet oxygen probe. Briefly, 1 mL of a CBM solution containing DPBF (30 μM) was irradiated for 360 s using 808 nm NIR light at 1 W/cm^2^. The absorbance values of DPBF at 424 nm and cypate at 786 nm were measured immediately.

### 2.9. In Vitro Drug Release Study

The drug release behaviors of DOX from DCBM were measured using the dialysis method in the presence or absence of external stimuli (GSH or NIR) [40]. The formulations were added in PBS (pH = 7.4) containing 10 mM or 30 mM of GSH. Then, the DOX release behaviors were performed at 37 °C. At the determined time points, 1 mL of sample was isolated from the release medium and the equivalent fresh medium refilled drug release system. The release behaviors in the presence of NIR light were carried out in PBS containing 10 mM of GSH, and other procedures were the same as the above introduction. A UV-Vis spectrometer was used to determine the concentration of DOX at 481 nm.

### 2.10. Cell Culture and Cellular Uptake

Cellular uptake of DCBM was determined by a confocal laser scanning microscope (CLSM), and flow cytometry was used to observe the intrinsic fluorescence of DOX. HepG2 cells were maintained in DMEM medium containing 10% FBS in an incubator with 5% CO_2_ at 37 °C. The cells were seeded onto a confocal dish and incubated with free DOX and DCBM (at an equal DOX concentration with 5 μg/mL) for 1, 2, and 4 h at 37 °C. Afterward, the cells were washed three times and fixed with 4% paraformaldehyde for 15 min. Cellular fluorescence was observed by CLSM. Flow cytometry measurements were conducted by living cell suspensions and then analyzed using a flow cytometer (Beckman, CA, USA).

### 2.11. Intracellular ROS Generation upon NIR Irradiation

The elevation of reactive oxygen species in HepG2 cells by DCBM micelles was measured using a DCFH-DA probe. Briefly, HepG2 cells were treated with DCBM (5 μg/mL of cypate) for 4 h and then washed with PBS three times to remove the free DCBM. After being exposed to NIR light (808 nm, 1 W/cm^2^) for 300 s, HepG2 cells were cultured with DCFH-DA (10 μM) for 20 min and washed with fresh medium. The same performance was carried out in the control group. The green fluorescence signal of DCFH-DA was measured using CLSM.

### 2.12. Cytotoxicity Evaluation and Apoptosis/Necrosis Detection

The cytotoxicity of DCBM was investigated using the CCK-8 assay. Briefly, HepG2 cells were seeded onto 96-well plates at a density of 6 × 10^3^ cells per well, and attachment to the culture vessel after incubating for 24 h was allowed. After that, HepG2 cells were incubated with blank micelles (cypate concentrations: 0, 1, 5, 10, 25 μg/mL) and DCBM (DOX concentration: 0, 1, 5, 10, 25 μg/mL; corresponding cypate concentrations: 0, 1, 5, 10, 25 μg/mL) in the presence or absence of NIR light (808 nm, 1 W/cm^2^, 300 s) after 12 h incubation and were further incubated for 24 h. Afterward, a CCK-8 kit was added, and the optical density (OD) was measured at 490 nm.

We determined the percentage of necrosis and apoptosis using flow cytometry. HepG2 cells grown in six-well plates were incubated with CBM and DCBM for 12 h, and after that, NIR irradiation (808 nm, 1 W/cm^2^, 300 s) was carried out. NIR absence was used for the control groups. After incubating for another 24 h, the cells were washed and suspended in a binding buffer, and the staining of these cells was carried out with Annexin V-FITC and propidium iodide (PI) for 15 min in the dark at room temperature. These samples were quantified by flow cytometry.

### 2.13. In Vivo IR Imaging and Anticancer Activity

C57BL/6 mice were subcutaneously inoculated with rat-derived Lewis lung cancer cells (1 × 10^6^). When the tumor volume reached approximately 50 mm^3^, the mice were used for an assay of photothermal imaging activity. The mice were injected with 100 μL of PBS and DCBM (5 μg/mL of cypate) through intratumor injection. The tumor sites were exposed to NIR laser (808 nm, 1 W/cm^2^, 300 s) as indicated 2 h post-injection. Temperature alterations in the tumor sites were recorded using an IR camera (FLIR T420). The tumor volume was calculated by the formula (mm^3^) length × width^2^/2. The animal experimental procedure was approved by the Animal Care Committee of Jilin University (License No.: 20160518) on 18 May 2016 and conformed to the Animal Ethical Standards and Use Committee at Jilin University.

For in vivo anticancer activity of DCBM, the C57BL/6 with lewis lung cell (LLC) tumor-bearing mice were evaluated. We randomly divided the mice into four groups (*n* = 3 per group), including DCBM plus NIR laser, DCBM only, CBM only, and PBS as the control group. We intravenously injected 100 μL of nanoparticles (DOX dose = 5 mg/kg, cypate dose ≈ 5 mg/kg) or PBS at 4, 6, and 8 days. The NIR + DCBM groups were irradiated with an 808 nm laser (1 W/cm^2^, 3 min) at 5 h post-injection. Tumor growth was monitored over 14 days.

## 3. Results and Discussion

### 3.1. Preparation and Characterization of Copolymers

**Figure 1:**
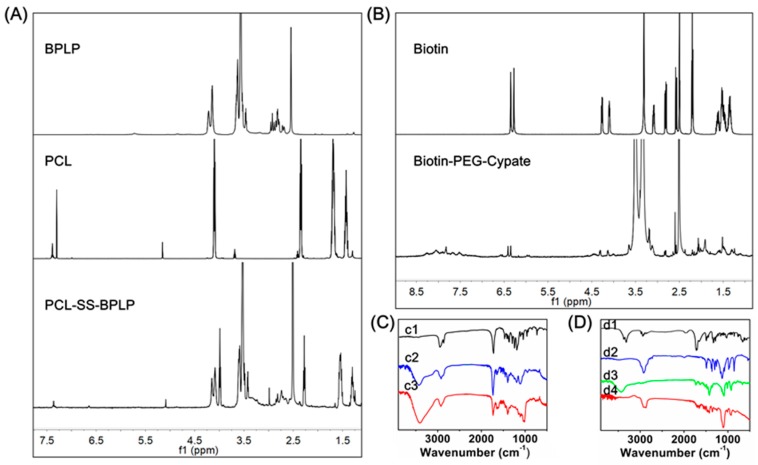
(**A**) ^1^H NMR spectra of PCL-SS-BPLP of DCBMs; (**B**) ^1^H NMR spectra of biotin-PEG-cypate of DCBMs; (**C**) FTIR spectra of PCL (c1), BPLP (c2), PCL-SS-BPLP (c3); (**D**) FTIR spectra of biotin (d1), PEG (d2), cypate (d3), biotin-PEG-cypate (d4).

Polycaprolactone-disulfide bond-biodegradable photoluminescent polymer (PCL-SS-BPLP) was prepared by high-temperature condensation reaction of three monomers (citric acid, cysteine, and polyethylene glycol) and further chemically linked with cystamine and polycaprolactone (PCL). The expected structure for PCL-SS-BPLP is shown in Scheme 4. ^1^H NMR spectra of PCL-SS-BPLP verified the successful incorporation of functional segments into the polymers (Figure 1A), as indicated by the appearance of signals located at 1.29 ppm, 1.53 ppm, 2.26 ppm, and 3.98 ppm, which were assigned to the protons of –CH_2_– from polycaprolactone. The signals from 2.60 to 2.86 ppm, 3.39 to 3.42 ppm, 4.08 ppm, and 4.14 ppm were attributed to the protons from BPLP segments. In addition, the ^13^C NMR spectrum of PCL-SS-BPLP is shown in Figure S2. The signals at 173 ppm were assigned to the –COO–, and the signals at 24.0 ppm, 24.8 ppm, 27.7 ppm, 33.2 ppm, and 63.4 ppm were assugned to the –CH_2_– in PCL pegments. The signal at 69.7 ppm belonged to the –O–CH_2_– of PEG in BPLP segments. The signals at 27.9 ppm, 50.8 ppm, and from 97.8 ppm to 165.8 ppm were assigned to a conjugation structure of BPLP segments. The ratio between hydrophilic and hydrophobic segments was the key factor of stability and morphology in the self-assembly system. Previous work showed that when the mass fraction of hydrophilic segments was around 70%, the polymers were able to self-assemble into spherical micelles [41]. The signal intensity ratio of characteristic proton peaks in PCL and biodegradable photoluminescent polymer (BPLP) segments was calculated to obtain the segment mass fraction. The two segment mass fractions could be calculated by I_(b)_/I_(a)_. I_(b)_ was the integration of one segment’s total protons, while I_(a)_ represented the integration of benzyl carbon protons. According to the ^1^H NMR spectra, the segment mass fraction between PCL and BPLP was about 2:5, and mass percentage of hydrophilic segments (BPLP) was close to 70%, meaning the polymers might have assembled into spherical micelles.

Biotin-polyethylene glycol-cypate (biotin-PEG-cypate) was prepared by chemically linking cypate with biotin-PEG2000-NH_2_. The expected structure of PCL-SS-BPLP is shown in Scheme 2. As shown in Figure 1B, the resonance peaks at 3.51 ppm were the –CH_2_CH_2_O– protons of polyethylene glycol (PEG), and peaks at 6.35 ppm, 4.31 ppm, and 4.14 ppm were ascribed to be protons from biotin. Moreover, the peaks at 5.95 ppm and 6.56 ppm were the double bond carbon protons of cypate, and from 7.33 to 8.35 ppm they were the aromatic carbon protons of cypate. The ^13^C NMR spectrum of biotin-PEG-cypate is presented in Figure S2: The signals at 162.6 ppm and 172.0 ppm belonged to the –CO– in polymer chains, and the peaks from 25.2 ppm to 28.0 ppm and from 55.4 ppm to 61.0 ppm might have belonged to –CH_2_– in biotin and –CH_3_ in cypate. The weak peaks around 128.8 ppm might have been assigned to the aromatic carbon and double bond carbon, and the signal at 69.8 ppm belonged to PEG in biotin-PEG-cypate. Thus, the appearance of targeted biotin and hydrophobic cypate fractions in the PEG chain implied the successful synthesis of biotin-PEG-cypate.

We further utilized elemental analysis and gel permeation chromatography (GPC) to investigate the structure of PCL-SS-BPLP and biotin-PEG-cypate. The approximate molecular formula of biotin-PEG-cypate was C_141_H_239_O_52_N_6_S, and the theoretical ratios of each element were 58.8%, 8.3% 2.9%, and 1.1%, corresponding to C, H, N, and S. In addition, the approximate molecular formula of PCL-SS-BPLP was (C_79_H_135_O_42.5_N_5_S_5_)_n_, according to the ^1^H NMR. The theoretical ratios of each element in PCL-SS-BPLP were 47.7%, 6.7%, 3.5%, and 8.0%, corresponding to C, H, N, and S. The measured values were generally close to the theoretical value in Table S1. The difference between measured values and theoretical mainly resulted from the non-uniformity and uncertainty of the polymer molecular composition. The molecular weights of the PCL-SS-BPLP polymer and biotin-PEG-cypate as measured by GPC were 13,428 g/mol and 3307 g/mol, respectively.

PCL-SS-BPLP and biotin-PEG-cypate polymers were also characterized by FTIR spectra. As illustrated in Figure 1C,D, the FTIR spectrum of PCL-SS-BPLP contained the characteristic bands of –CONH– at 3426 cm^−1^ and 1635 cm^−1^. The peak at 1731 cm^−1^ corresponded to the –CO– stretching vibration. The transmission peak around 2930 cm^−1^ was attributed to the –CH_2_– stretching vibration. The peaks of PCL were overlapped by BPLP segments. The FTIR spectrum of the biotin-PEG-cypate that contained the characteristic band of –CO– stretching at 1699 cm^−1^ and the transmission peaks of 2901 cm^−1^ and 1102 cm^−1^ belonged to the CH_2_–CH_2_–O– of biotin-PEG-cypate.

### 3.2. Preparation and Characterization of Mixed Micelles

**Figure 2:**
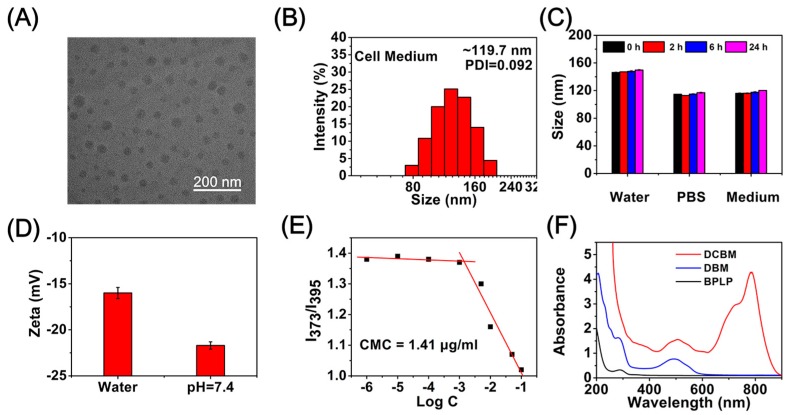
(**A**) TEM images of DCBMs; (**B**) DLS distribution of DCBMs in cell medium; (**C**) size stability of DCBMs in water, phosphate-buffered saline (PBS), and cell medium at 24 h; (**D**) zeta potential of DCBMs in pure water and pH = 7.4 aqueous solution; (**E**) the plot of the ratio of intensities (I373/I395) from pyrene emission spectra as a function of log C; (**F**) UV spectra of BPLP, DBMs, and DCBMs in PBS.

Doxorubicin/cypate-loading biodegradable micelles (DCBMs) were prepared by a dialysis method, mixing two polymers and DOX proportionally. TEM micrographs and size distributions of mixed micelles are presented in Figure 2A. TEM results revealed the spherical mixed micelles were constructed in a dry condition with a size of ~50 nm. In addition, the hydration diameter of DBCM in cell medium was approximately 119.7 nm (Figure 2B) with a narrow distribution (PDI) of 0.092. Zeta potentials were about −16.0 mV in water and −21.7 mV at pH 7.4 (Figure 2D). To investigate the size stability of DCBM in water, PBS, and cell medium, we performed DLS measurements at times of 0 h, 2 h, 6 h, and 24 h (Figure 2C). The DLS results revealed that no obvious size alternation appeared in a specified solvent with an increase in incubation time. Meanwhile, the smaller sizes in PBS and cell medium might be attributed to the stronger negative charge on the DCBM shell, improving the electrostatic repulsion among polymer chains and further making fewer polymer segments assemble into the micelles. These DLS and zeta potential results indicated the DCBM could keep stability in a physiological environment. The collective micellization behaviors of PCL-SS-BPLP and biotin-PEG-cypate could be confirmed by measuring the critical micelle concentration (CMC) value using pyrene as a hydrophobic fluorescence probe. As the curve presents in Figure 2E, the CMC of the mixed micelles was at a relatively low level (1.41 μg/mL), indicating their excellent self-assembly ability in aqueous solution and the potential ability for their utilization in a physiological environment [36]. The UV spectra of DCBM showed absorption peaks (Figure 2F) at 786 nm (belonging to cypate), 485 nm (belonging to DOX), and 284 nm (belonging to BPLP), indicating that DCBMs were successfully synthesized and incorporated DOX and cypate into DCBMs.

### 3.3. Degradable Properties

**Figure 3:**
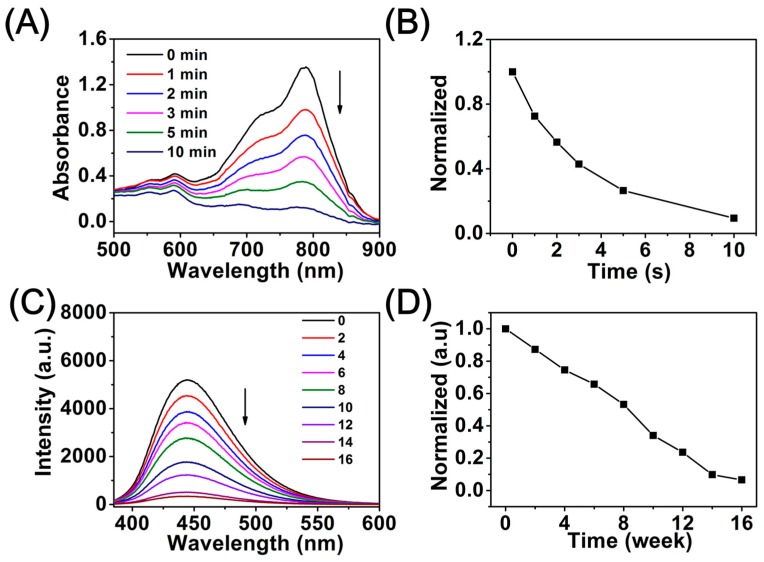
(**A**) Time dependence of absorption of DCBMs under NIR excitation (808 nm, 1 W/cm^2^, 10 min); (**B**) normalized absorption of cypate in the DCBM solutions as a function of irradiation time; (**C**) photoluminescence emission spectra of degradation solutions of PCL-SS-BPLP; (**D**) normalized curves corresponding to C.

The decomposition property of cypate in the biotin-PEG-cypate polymer was measured by a UV-Vis spectrophotometer to demonstrate the disassembly profiles of the micelles (Figure 3A,B), which was carried out by a continuous-wave NIR light (808 nm, 1 W/cm^2^). With the increase of NIR irradiation time, the absorption peak value at 786 nm gradually decreased, and only 9.5% of maximum absorption intensity could be observed at 10 min post-irradiation. Cypate underwent a consumptive, decomposable, and irreversible process under NIR irradiation, and NIR-triggered destruction of the cypate chromophore structure presumably induced rapid photodecomposition and photobleaching, leaving behind more hydrophilic PEGylated fragments, breaking the balance stability of the mixed micelles, thereby resulting in the rapid release of DOX from micelles [27]. Previous work revealed that the degradation products of BPLP were less toxic, which was a key factor for applications in clinical therapy [31]. Additionally, the degradation property of the PCL-SS-BPLP copolymer was conducted in PBS solution, and the photoluminescence emission in the process of PCL-SS-BPLP degradation was tracked every two weeks to investigate the time-dependent degradation ability of BPLP segments. The corresponding fluorescence signal loss is shown in Figure 3C: During 16 weeks of degradation of PCL-SS-BPLP, the fluorescence emission intensity of the PCL-SS-BPLP copolymer decreased with increasing degradation time, and the emission peaks were observed at a constant wavelength of 444 nm. As shown in Figure 3D, the normalized deviation value of fluorescence intensity was about 0.033 at the degradation period between the 14th week and the 16th week, which was much smaller than that of the previous degradation period (deviation value = 0.134). It was obvious that the BPLP segments attained a nearly complete degradation within 16 weeks. These results indicated that inherent and stable photoluminescence of PCL-SS-BPLP can be preserved for almost the entire physiological action time, and BPLP segments of PCL-SS-BPLP copolymer are degradable in a physiological environment to achieve safe and nontoxic drug delivery.

### 3.4. Photoresponsive Effects and ^1^O_2_ Generation

**Figure 4:**
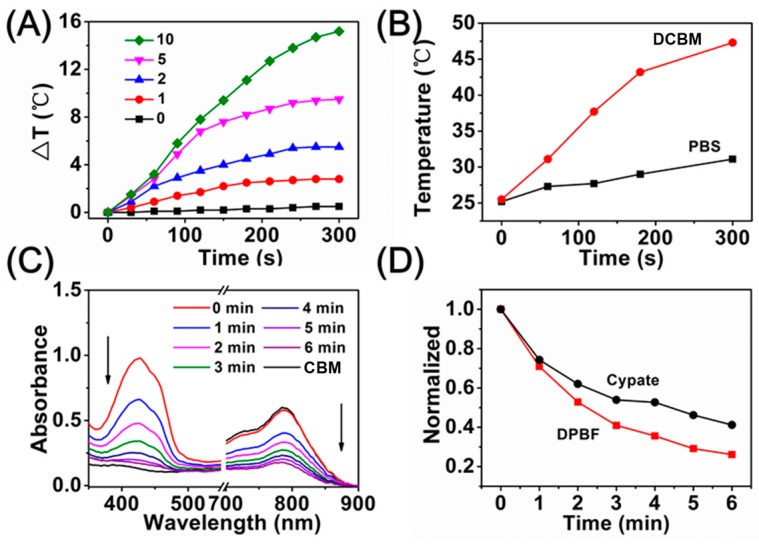
(**A**) Temperature change of DCBMs containing various concentrations of cypate (μg/mL) under 808 nm irradiation (1 W/cm^2^); (**B**) temperature curves of the IR image groups; (**C**) extracellular reactive oxygen species (ROS) generation of different formulations under irradiation at 808 nm for 6 min using 1,3-disphenylisobenzofuran (DPBF) as an indicator; (**D**) normalized absorption of DPBF in the DCBM solutions as a function of irradiation time.

Generally, cyanine derivatives possess photothermal conversion efficiency to varying degrees at the strong absorbance area [39]. Therefore, the strong absorbance of these micelles at 808 nm (from the PEGylated cypate) suggested that the micelles possessed a photothermal conversion ability. The photothermal conversion efficiency of free cypate was about ~11.6% in water/ethanol = 9:1, while DCBM was determined to be ~14.9%, indicating the DCBMs could improve the thermal effect of cypate in anticancer procedures (Figure S6). The DCBM solution with various concentrations was irradiated by NIR light (808 nm, 1 W/cm^2^) for 300 s. The DCBM solution could quickly generate hyperthermia, and a more rapid temperature increase was observed for higher concentrations. As shown in Figure 4A, the DCBMs exhibited a remarkable temperature elevation (ΔT) from 2.8 to 15.2 °C upon NIR exposure within 300 s, containing the cypate concentration range of 1–10 μg/mL. The surface photothermal performance of DCBM was monitored by IR thermal imaging (Figure S4) and further converted to a temperature change curve (Figure 4B), which corresponded with an internal temperature change. This indicated that the mixed micelles exhibited a concentration-dependent temperature increase and played a significant role in cancer cell damage (above 42 °C). To investigate the PDT profile of the DCBMs, singlet oxygen (^1^O_2_) generation was evaluated using 1, 3-diphenyl isobenzofuran (DPBF) as a specific probe. As shown in Figure 4C,D, the DCBM samples exhibited a gradual decline in DPBF absorbance at 424 nm under NIR light exposure for 6 min, suggesting the sustained generation of ^1^O_2_. Reasonably, concomitant absorbance (at 786 nm) decline was synchronous to the absorbance change of DPBF within the irradiation time, demonstrating that the decomposition of cypate accompanied the generation of ^1^O_2_ [27]. These results revealed that DCBMs could significantly generate singlet oxygen under NIR light irradiation, which can be potentially applied in clinical cancer for phototherapy.

### 3.5. In Vitro Drug Release Study

**Figure 5:**
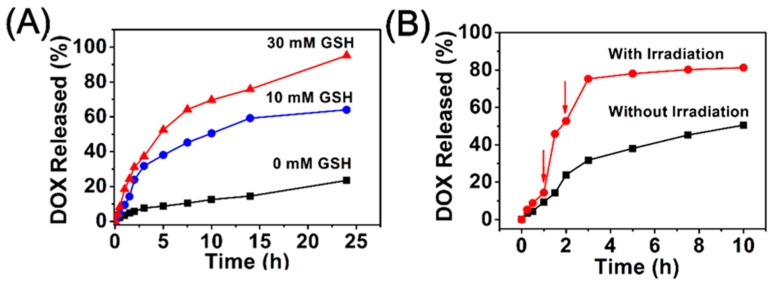
(**A**) Accumulative release profiles of DOX from DCBMs at different concentrations of GSH; (**B**) accumulative release profiles of DOX from DCBMs at 10 mM of GSH with or without NIR irradiation.

The release behavior of DOX from the DCBMs was determined by a dialysis method in the presence or absence of external stimuli (GSH and NIR) [40]. The DOX content of micelles was about 10.7%. As shown in Figure 5A, after 24 h dialysis at pH 7.4 in various concentrations of GSH, the cumulative release was only 23.5% without GSH, resulting from DOX’s tiny premature leakage from DCBMs. In contrast, a rapid release of DOX was about 64.0% after the addition of 10 mM GSH to the PBS due to the reduction of sensitive disulfide bond scission in DCBMs. Moreover, 95.1% of DOX loaded in the micelles was released after the addition of 30 mM GSH. These results verified that the cumulative drug release was GSH concentration-dependent. Next, the NIR light-mediated release behavior of DOX was investigated with 10 mM of GSH. As depicted in Figure 5B, when the DCBM was irradiated with NIR light (808 nm, 1 W/cm^2^) for 300 s at the preconcerted time point (1 and 2 h), the accumulative DOX release property showed an accelerative release pattern at the next sampling time point (45.8% and 75.3%, at 1.5 h and 3 h), compared to that of NIR absence (14.2% and 31.8%). These results indicated that the rapid DOX release from DCBMs could be triggered by NIR light, which could be attributed to cracking of the micelles by NIR-mediated cypate decomposition and weakening of the hyperthermia-mediated electrostatic interactions between DOX and micelles.

### 3.6. Cellular Uptake

**Figure 6:**
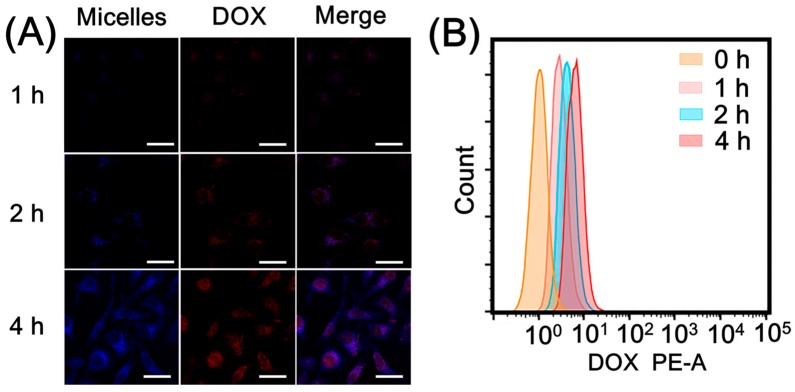
(**A**) Confocal laser scanning microscope (CLSM) images of HepG2 cells after incubation with DCBMs for 1 h, 2 h, and 4 h. DOX exhibited red fluorescence, and BPLP segments showed blue fluorescence. The scale bars correspond to 30 μm in all the images. (**B**) Flow cytometry analysis of cellular uptake.

Cellular uptake behavior of DCBMs was evaluated by HpeG2 cells, and its procedure was monitored by CLSM. As shown in Figure 6A, the HepG2 cells were treated with DCBMs for different durations. Obviously, red fluorescence from DOX and blue fluorescence from BPLP segments were hardly observed at 1 h, and both fluorescences were much stronger and mostly overlapping in the cytoplasm at 2 h. The results indicated that cells could take up DCBMs through endocytosis, and only tiny amounts of DOX were released from DCBMs. Meanwhile, the intensive fluorescence of DOX after 4 h incubation was observed in the cytoplasm and part of the fluorescence in the cell nucleus, but the blue fluorescence could not be seen in the nucleus, which could be attributed to GSH-induced cleavage of DCBMs. The cellular uptake of free DOX (Figure S5) was quicker than the DCBM group. These results implied that the increased accumulation of the micelles in the endocytosis followed by the prolonged incubation time and the accumulative release of DOX could penetrate the nucleus to induce cell apoptosis. Flow cytometry quantitatively analyzed cell uptake consisting of the CLSM observation. In the DCBM group, the intracellular DOX fluorescence of HepG2 cells gradually became strong (Figure 6B) within the incubation time, corresponding to the cellular uptake behavior in CLSM.

### 3.7. Intracellular ROS Generation upon NIR Irradiation

**Figure 7:**
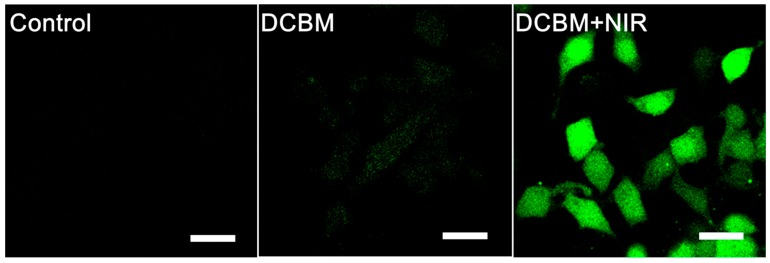
CLSM images of ^1^O_2_ generation in HepG2 cells after incubation with DCBMs for 4 h. The scale bars correspond to 30 μm in all the images.

DCFH-DA, a fluorescence probe for intracellular reactive oxygen species detection, was used to detect the light-driven singlet oxygen (^1^O_2_) generation and PDT effect of DCBMs [42]. Figure 7 displays the mean fluorescence intensity of DCBMs in the presence or absence of NIR light irradiation and the PBS treatment group as a control. It was obvious that no fluorescence signal could be observed in the PBS group without NIR light irradiation, suggesting that ^1^O_2_ could not be produced in a normal physiological environment. In addition, strong green fluorescence intensity was displayed in the group of DCBMs with NIR light irradiation (808 nm, 1 W/cm^2^, 300 s), whereas very weak green signals could be observed without NIR-mediated treatment. These results indicated that DCBMs could be internalized by cancer cells without significant ^1^O_2_-mediated cytotoxicity in the absence of NIR light and could generate plenty of ^1^O_2_ for simultaneous PDT therapy in the presence of NIR light.

### 3.8. Cytotoxicity Evaluation

**Figure 8:**
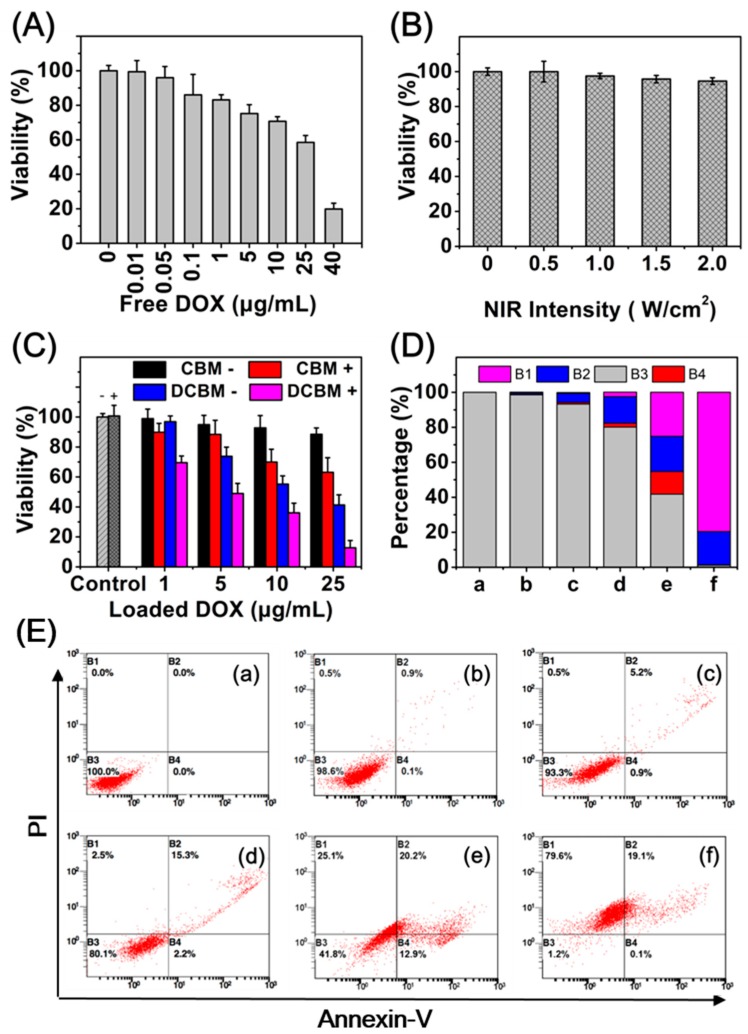
(**A**) Cell viability of HepG2 cells treated with free DOX for 24 h; (**B**) Cell viability within 24 h under different intensity NIR irradiation only for 5 min; (**C**) Cytotoxicities of blank micelles (CBMs), DOX-loading micelles (DCBMs), and the control group with/without laser irradiation (+/−) in HepG2 cells after 24 h treatments using the CCK-8 assay; (**D**) Percentages of cells with different apoptosis analysis stages by flow cytometry. B1: Necrosis; B2: Late apoptotic; B3: Early apoptotic; B4: Viable; a: Control; b: Control + NIR; c: CBM; d: CBM + NIR; e: DCBM; f: DCBM + NIR; (**E**) Apoptosis analyzed by flow cytometry in HepG2 cells at 24 h after various treatments.

To examine the synergistic cytotoxicity of DCBMs against cancer cells, we incubated HepG2 cells for 24 h with free DOX, only cypate-loading biodegradable micelles (CBMs) and DOX-loading micelles (DCBMs) after NIR irradiation (5 min) with different intensities. The HepG2 cells without co-incubation were a control group. HepG2 cells exhibited a DOX dose-dependent cytotoxicity in Figure 8A. As shown in Figure 8B, NIR irradiation of different intensities (from 0.5 to 2 W/cm^2^) had negligible cytotoxicity, indicating the safety of NIR irradiation. In Figure 8C, CBM exhibited negligible cytotoxicity in the absence of NIR, indicating weak potential toxicity of CBM micelles as well as the loaded cypate. On the contrary, the viabilities of HepG2 cells gradually declined from 89.8% to 63.1% after NIR light irradiation, showing cypate concentration-dependent cytotoxicity. In the DOX-loading groups, the viability of DCBMs plus NIR decreased from 69.6% to 12.7%, displaying the best antitumor effects among the free DOX group, the DCBM-only group, and the CBM + NIR group at the same drug dose level. This phenomenon might be attributed to cell dual damage from chemotherapy and phototherapy. Thus, synergistic chemophototherapy of DCBM was highly effective on HepG2 cells.

Flow cytometry was performed with annexin-V/PI staining assay to quantitatively determine cell apoptosis after different treatments. As shown in Figure 8D,E, compared to the control group (a), NIR irradiation alone only (b) slightly induced necrosis and apoptosis, suggesting that NIR irradiation hardly induced cell apoptosis due to its weak phototoxicity. In the blank micelles (CBMs) groups, about 17.8% necrosis cells and apoptosis cells were observed in the presence of NIR (d), notably higher than the NIR absence group (c), indicating the obvious PDT and PTT efficiencies of cypate-loading micelles. Furthermore, about 45.3% apoptosis and necrosis cells were obtained in the DCBM group (e), and the cell death rate was much more significant under NIR irradiation (f), displaying an excellent chemophototherapy ability of DCBMs.

### 3.9. In Vivo IR Imaging and Anticancer Activity

**Figure 9:**
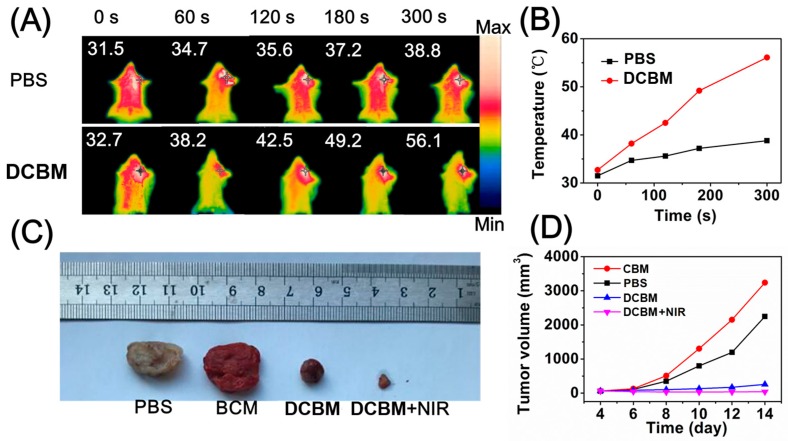
(**A**) In vivo photothermal effects of DCBMs; (**B**) temperature curves of the NIR-irradiated groups; (**C**) photographs of ex vivo tumor tissue after 14 days of treatment; (**D**) corresponding tumor inhibition rate recorded after administration from 4 to 14 days.

Encouraged by the in vitro photothermal behavior of DCBM micelles, we further carried out in vivo temperature changes on the LLC-bearing mice. After 2 h intratumoral injection with PBS and DCBM, the tumor site was exposed to 808 nm laser (1 W/cm^2^) for 300 s, followed by recording the temperature alterations at the indicated time points. As depicted in Figure 9A,B, the tumor surface temperature of the DCBM-treated group rapidly increased to 56.1 °C (Δ*T* = 23.4 °C) during the irradiation process, which was enough to damage the tumor tissue (the thermal-induced cancer cell inactivation temperature was higher than 42 °C). On the contrary, the temperature of the PBS-treated group increased to 38.8 °C (Δ*T* = 7.3 °C), which was not enough to kill cancer cells. These results revealed that DCBMs could efficiently convert NIR energy to hyperthermia in vivo.

The in vivo anticancer efficacy of the DCBMs was studied on the C57BL/6 LLC-bearing mice in combination with NIR irradiation. The mice were treated by intravenous injection, accompanied by NIR irradiation (808 nm, 1 W/cm^2^, 3 min) at 5 h post-injection. The volume of the tumor during the DCBM + NIR synergistic therapy process is shown in Figure 9D. Treatment with three injections of DCBMs inhibited the growth of LLC tumors and was followed by NIR irradiation every time. Significantly, the anticancer effect of DCBMs was enhanced when combined with the NIR irradiation (Figure 9C). These preferable tumor inhibition effects of DCBM + NIR due to dual phototoxicity of ROS-induced damage from PDT and hyperthermia-induced damage from PTT, compared to the DCBM-only group. Moreover, the CBM group showed the largest tumor tissue compared to other groups, attributed to the nontoxicity of blank micelles. The in vivo results indicated that DCBMs were an effective drug delivery system to apply in tumor ablation.

## 4. Conclusions

In summary, doxorubicin/cypate-loading biodegradable micelles (DCBMs) were developed for cancer chemo/PTT/PDT therapy. The DCBMs possessed a relative stability and a hydration diameter (~119.7 nm) in cell medium, low critical micelle concentration, and a narrow distribution. They also could target cancer cells and transport DOX to tumor cytoplasm and rapidly release DOX in cell cytoplasm with a high concentration of GSH and NIR-mediated cypate composition. The potential degradation ability and photocomposition ability of DCBMs suggested the micelles could be degraded in a physiological environment. Particularly, the soluble PEGylated cypate improved the utilization rate of cypate, and PDT/PTT from cypate accelerated cell death in vitro and tumor ablation in vivo. These DCBMs not only potentially possess a degradation ability, but also efficiently exerted trinitarian synergism of photothermal/photodynamic/chemotherapies, resulting in an excellent antitumor effect. The presented micelles provide a versatile synergistic strategy for enhanced anticancer therapy.

## Supplementary Materials

Supplementary materials can be found at http://www.mdpi.com/2079-4991/9/1/91/s1, Table S1: Elemental analysis and gel permeation chromatography (GPC) of PCL-SS-BPLP and biotin-PEG-cypate, Figure S1: ^1^H NMR spectrum of BPLP in D_2_O, Figure S2: ^13^C NMR spectrum of PCL-SS-BPLP and biotin-PEG-cypate in DMSO-d_6_, Figure S3: Emission and excitation spectra of PCL-SS-BPLP, Figure S4: The surface photothermal performance of DBCM, Figure S5: Confocal image of HepG2, the red fluorescence is from DOX. Scale bars = 50 µm, Figure S6: Photothermal heating and cooling curves of the DCBM, free cypate and solvent (water: ethanol = 9:1) under NIR laser irradiation, followed by switching of the laser.
