# Supplementary material for: Biodegradable Micelles for NIR/GSH-Triggered Chemophototherapy of Cancer
Source: Nanomaterials (Basel). 2019 Jan 11;9(1):91. doi: 10.3390/nano9010091 (PMC6359036; doi:10.3390/nano9010091)
Supplement: Supplementary file 1 [file nanomaterials-09-00091-s001.pdf]

Supplementary Materials

Biodegradable Micelles for NIR/GSH-Triggered Chemophototherapy of Cancer

Table S1. Elemental analysis and gel permeation chromatography (GPC) of PCL-SS-BPLP and biotin-PEG-cypate.

	Elemental analysis				GPC		
	C	H	N	S	M_n	M_w	PDI
PCL-SS-BPLP	2.5%	54.9%	6.9%	5.6%	9135	13428	1.47
Biotin-PEG-cypate	4.8%	54.8%	7.7%	0.9%	2576	3307	1.28

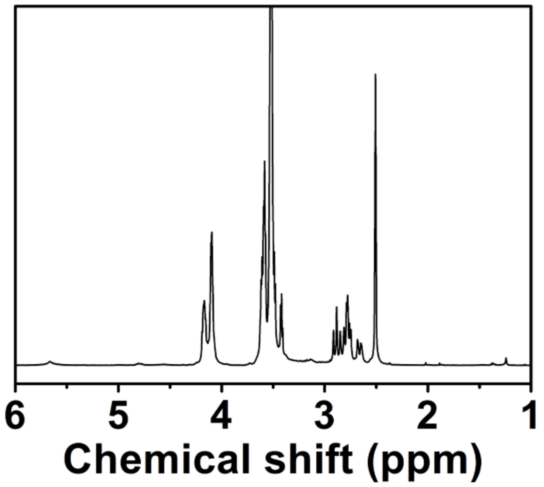

Figure S1. ^{1}H NMR spectrum of BPLP in D_2O.

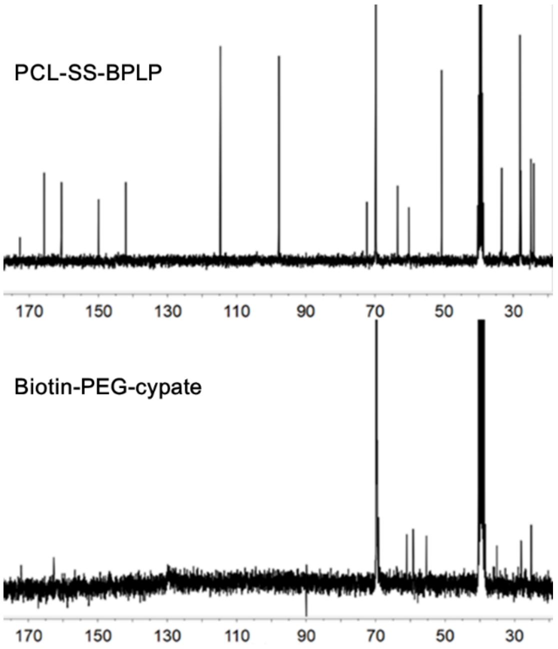

Figure S2. ^{13}C NMR spectrum of PCL-SS-BPLP and biotin-PEG-cypate in DMSO-d_6.

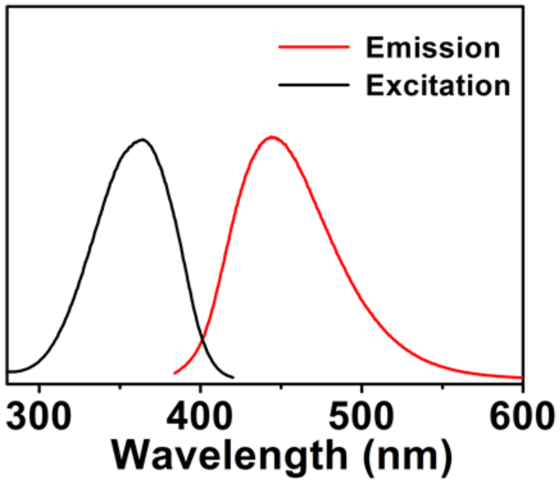

Figure S3. Emission and excitation spectra of PCL-SS-BPLP.

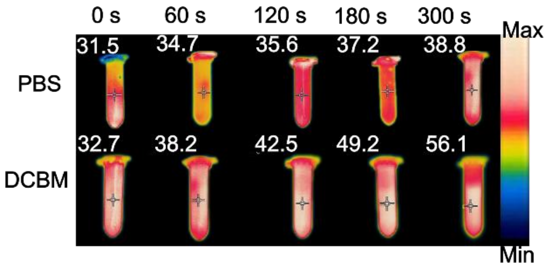

Figure S4. The surface photothermal performance of DBCMs.

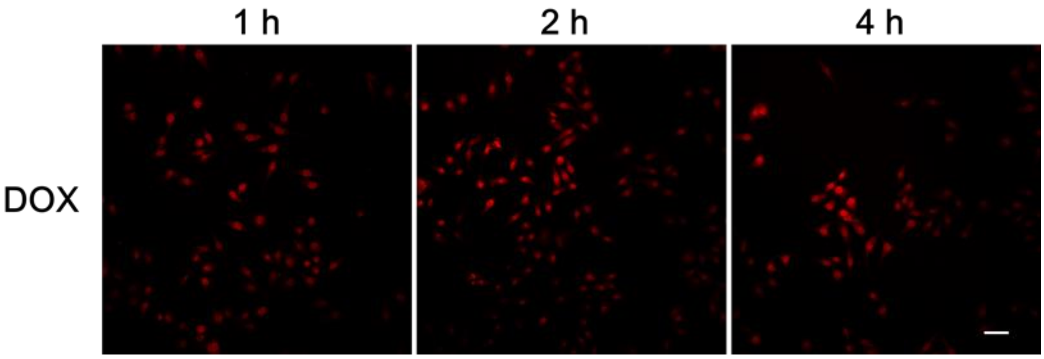

Figure S5. Confocal image of HepG2. The red fluorescence is from DOX. Scale bars = 50 μm.

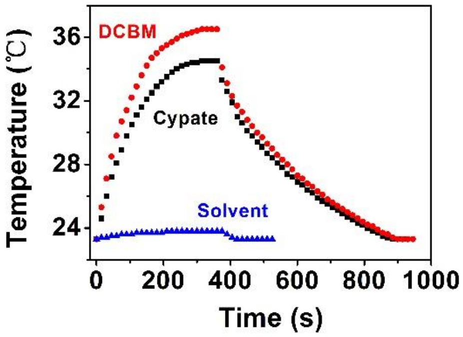

Figure S6. Photothermal heating and cooling curves of the DCBMs, free cypate, and solvent (water/ethanol = 9:1) under NIR laser irradiation, followed by switching off the laser.
